# Costing universal health coverage

**DOI:** 10.2471/BLT.19.229799

**Published:** 2019-09-30

**Authors:** Joseph Wong, Kimberly Skead

**Affiliations:** a Munk School of Global Affairs and Public Policy, University of Toronto, 1 Devonshire Place, Toronto, M5S 3K7 Ontario, Canada.; b Department of Molecular Genetics, University of Toronto, Toronto, Canada.

The sustainable development goals (SDGs) are audacious and clear. SDG 1 aims to eliminate poverty in all its forms everywhere, meaning that no one is to be left behind. SDG 3 focuses on health and well-being and targets to achieve universal health coverage (UHC) by 2030. However, health – and development interventions in general – must reach everyone if these goals are to be achieved.

To ensure that no one is left behind, we need to reach the hardest-to-reach: the poorest of the poor, the geographically isolated, the administratively invisible, those without identification documents or an address, and the marginalized. Achieving UHC requires meeting the challenge of delivering services to those most in need and more specifically to those who are hardest-to-reach.^1^

The Reach Project at the University of Toronto is investigating how reach can be achieved.^2^ The project is a collaborative and multidisciplinary research programme, in which faculty researchers from the social sciences, management, medicine, public health and engineering, work together with students to examine how health and development interventions have successfully reached the hardest-to-reach in low- and middle-income settings. Over the past four years, researchers in the project have conducted archival research and fieldwork in several countries, looking at a range of important case studies including vaccine delivery to rural populations in India and Rwanda; the implementation of birth registration schemes in South Africa; the delivery of cash transfers in Brazil, Jordan and West Bank and Gaza Strip; the elimination of mother-to-child-transmission of human immunodeficiency virus in remote areas of Thailand; and many more.^2^

The case studies offer important insights for governments, nongovernmental organizations, firms and international donors. These studies include: the pivotal role that effective monitoring plays in reaching the hard-to-reach; creative efforts to accurately identify and locate the poorest of the poor; the generation of political will to overcome legacies of mistrust and marginalization among certain population segments; the importance of mobile units and digital platforms to actively reach those who live far from urban centres; the interaction of computer mapping technology and local knowledge to facilitate route optimization for cold-chain delivery of essential medicines; and the critical role played by community health workers, and specifically the balance between coordinated protocols with adaptive local knowledge so that frontline workers can deliver interventions in local contexts.

Reaching the hard-to-reach demands innovative delivery mechanisms, but also attention to the economic and financial cost of reaching the previously unreached, an observation raised by the World Health Organization (WHO) in its 2014 *Making Fair Choices on the path to universal health coverage* report.^3^ Distance to travel and time, for example, dramatically affect the cost of delivering health interventions to remote places. Personnel and training costs of delivering health interventions to sparsely populated and distant regions can be extremely high. Maintaining an accurate registry of poor households, which is often used in targeted health programmes, is difficult and costly.^4^

In other words, ensuring no one is left behind is expensive. Indeed, if the costs become too high, such initiatives become prohibitively expensive, in which case governments fail to generate the political will to reach the hard-to-reach and nongovernmental organizations cannot devise a feasible delivery model. The challenges of making a case to reach the hardest

-to-reach are compounded further when we consider the difficulties of estimating the economic returns generated by reaching the hardest-to-reach especially in terms of the impact of health interventions. Simply put, when costs become too high and when returns are unclear, many will remain unreached and thus be left behind. 

Cost is an important variable and health and development research has a relatively good handle on aggregate cost data. Governments, development agencies and nongovernmental actors should know how much it costs to deliver health and development interventions. A good example is the United States Agency for International Development Deliver Project in United Republic of Tanzania. The Medical Stores Department of the country, a semi-autonomous public agency that oversees the procurement and distribution of medicines, uses mapping software to optimize routes for transport trucks to deliver vaccines and medicines to over 5000 clinics.^5^ Route optimization involves calculating the most effective (accurate and on-time delivery) and cost–efficient (distance and time travelled) delivery routes, affecting the programme’s total cost.

Total cost is an important measure because we can assess the overall amount of resources needed to deliver, for example, vaccines and medicines to all clinics. Total cost also allows a calculation of the average cost to deliver on a per-capita (or per-clinic) basis. However, we contend that it is more important to know the marginal cost than total cost to deliver interventions as coverage expands. Marginal cost is the additional resources required to deliver an intervention to an additional beneficiary or clinic. 

In general, we expect that as coverage rates expand marginal cost will rise more steeply compared to average cost. This increase is opposite to the conventional wisdom that costs should decline as markets expand because of the economies of scale.^6^ Marginal costs rise as we reach the hardest-to-reach because populations become sparser, the terrain becomes more difficult to travel, distances become longer and personnel costs increase, among other factors. Unlike average cost, changes in marginal cost are not linear, with significant implications. A recent report points out that overreliance on total or average cost in determining aid allocation underappreciates the higher marginal costs of ensuring development aid is distributed to the poorest. Most aid tends to reach those who live in denser and thus urban areas because such approach is overall more cost–effective, providing more value for money.^7^

Economic models can project the effect of coverage expansion on marginal cost. A study found that average and marginal cost to deliver a WHO outreach intervention in the Americas tracks relatively closely until about the 50% coverage rate.^8^ After 50% coverage, marginal and average cost begin to diverge, with marginal cost rising more severely versus average cost at around the 75% coverage rate. Marginal cost rises steeply again compared to average cost between 90 to 100% (or universal) coverage rates. At universal coverage, according to the model, marginal cost is calculated to be nearly 2.5 times the average cost of delivery over the entire programme.^8^

The rapid and steep rise in marginal cost means that it is difficult to generate the needed political will or market model to reach the hardest-to-reach. From a government’s point of view, reaching the hard-to-reach may not be politically worthwhile, just as a private sector actor or a nongovernmental organization might conclude that the market to reach those who are hardest to reach is simply not viable. Knowing the marginal cost increase to deliver essential health services to the hard-to-reach is critical in designing and implementing health and development interventions. Identifying which factors are driving marginal cost up so steeply is important to formulate solutions and innovate from a more robust evidence-base.

For example, Optima Nutrition is a tool that integrates marginal cost into cost modelling for multiple nutrition interventions to develop guidelines that optimize the impact of nutrition-related investments. An empirical study evaluated the allocation of resources for nutrition-related interventions in seven districts in Bangladesh, and found that marginal costs rise quickly and in a nonlinear fashion, meaning that the incremental gain per additional dollar spent decreased. According to the data from that study, the resources required to deliver the nutrition interventions increase considerably to cover the marginal cost of coverage expansion.^9^ Additional tools such as Marginal Budgeting for Bottlenecks identify implementation constraints of health service delivery and generate estimates of the marginal cost of overcoming these constraints to increase the coverage and quality of high impact interventions.

Both tools highlight the importance of incorporating marginal cost into cost models to optimize service delivery with constrained resources. Without an empirical understanding of how marginal costs change with changes in coverage, interventions might not reach their target populations and specifically those who stand to benefit from them the most. 

Calculating marginal cost remains elusive, however, particularly as interventions approach complete coverage. For the most part, cost modelling tools rely heavily on simulated data, as longitudinal information on cost and coverage is limited. Data collection tends to capture an aggregate snapshot, which includes both the variable and fixed costs at a specific moment in time, or after a programme has been rolled out. Since marginal cost is the amount needed to produce and deliver one additional unit, accurately calculating marginal cost demands new data, new modes of data collection and new assumptions. Specifically, the UHC research agenda must collect cost data serially, as coverage expands, and we should not presume changes in cost will necessarily follow a linear pattern. This way, we will be able to better identify marginal cost drivers, and in turn optimize delivery and implementation schemes to maximize the reach of health and development initiatives.
